# Copy number variations in primary tumor, serum and lymph node metastasis of bladder cancer patients treated with radical cystectomy

**DOI:** 10.1038/s41598-020-75869-x

**Published:** 2020-12-09

**Authors:** Armin Soave, Lan Kluwe, Hang Yu, Michael Rink, Philipp Gild, Malte W. Vetterlein, Philipp Marks, Guido Sauter, Margit Fisch, Christian P. Meyer, Tim Ludwig, Roland Dahlem, Sarah Minner, Klaus Pantel, Bettina Steinbach, Heidi Schwarzenbach

**Affiliations:** 1 Department of Urology, University Medical Center Hamburg-Eppendorf, Hamburg, Germany; 2 Department of Neurology, University Medical Center Hamburg-Eppendorf, Hamburg, Germany; 3 Institute of Pathology, University Medical Center Hamburg Eppendorf, Hamburg, Germany; 4 Department of Tumor Biology, University Medical Center Hamburg-Eppendorf, Martinistraße 52, 20246 Hamburg, Germany

**Keywords:** Cancer, Molecular biology, Urology

## Abstract

The aim of the present study was to analyze copy number variations (CNV) of multiple oncogenes and tumor suppressor genes in genomic DNA from primary tumor tissue, lymph node metastasis and cell-free DNA (cfDNA) from serum of 72 urothelial carcinoma of bladder (UCB) patients treated with radical cystectomy (RC), using multiplex ligation-dependent probe amplification (MLPA). We hypothesized that primary tumor and lymph node metastasis show similar CNV profiles, and CNV are more present in lymph node metastasis compared to primary tumor tissue. Samples from 43 (59.7%) patients could be analyzed. In total, 35 (83%), 26 (68%) and 8 (42%) patients had CNV in primary tumor, serum and lymph node metastasis, respectively. MYC, CCND1, ERBB2 and CCNE1 displayed the most frequent amplifications. In particular, CNV in ERBB2 was associated with aggressive tumor characteristics. CNV in both ERBB2 and TOP2A were risk factors for disease recurrence. The current findings show that CNV are present in various oncogenes and tumor suppressor genes in genomic DNA from primary tumor, lymph node metastasis and cfDNA from serum. CNV were more present in genomic DNA from primary tumor tissue compared to cfDNA from serum and genomic DNA from lymph node metastasis. Patients with CNV in ERBB2 and TOP2A are at increased risk for disease recurrence following RC. Further studies are necessary to validate, whether these genes may represent promising candidates for targeted-therapy.

## Introduction

Urothelial carcinoma of the bladder (UCB) is the second leading genitourinary cancer and a potentially lethal malignancy, with an incidence of over 80,000 new cases and over 17,000 estimated deaths in 2019 in the United States^1^. Radical cystectomy (RC) with bilateral pelvic lymphadenectomy represents the golden standard surgical treatment for muscle-invasive and recurrent high-risk non-muscle invasive UCB^2^. Outcomes have remained stable over the past decades^3^, and a relevant number of patients experience disease recurrence and progression within 2 years after RC^4^. Various clinic-pathologic UCB features and biomarkers have been investigated to allow identifying those patients, who are at the highest risk of suffering from poor outcome^5^. Genetic analyses, including copy number variations (CNV), have the potential to elucidate the cellular mechanisms involved in the pathogenesis of UCB, and by offering targets to emerging targeted-therapy may contribute to improve outcome.

With the emergence of high-throughput genomic profiling methods like next generation sequencing, the genomic landscape of UCB has come into focus of research. Comprehensive molecular characterization by The Cancer Genome Atlas (TCGA) revealed specific RNA and DNA alterations, such as CNV in UCB^6^. DNA deletions, insertions and duplications lead to CNV ranging in size from several dozens of bases to megabases. They either exhibit no phenotypic effect and implicate adaptive traits, or cause severe diseases. It has been estimated that up to 9.5% of the genome accounts for CNV and surprisingly, approximately 100 genes can be completely deleted without generating apparent phenotypic consequences indicating that these genes may be functionally redundant^7^. CNV are a hallmark of different cancer types, including UCB, and affect the activity of tumor-associated signaling pathways and anticancer drug sensitivity as well as toxicity^8^. CNV can be investigated in different sources, such as tissue and liquid biopsies, by different techniques, including array comparative genomic hybridization (array-CGH)^9^, droplet digital PCR^10,11^, whole genome sequencing^12^ and multiplex ligation-dependent probe amplification (MLPA)^13^.

To date, most studies have analyzed CNV in genomic DNA derived from tissues and only few studies in cell-free DNA (cfDNA) derived from plasma or serum^14,15^, because of its low quantity and quality. CfDNA is released into the blood circulation by cell death (apoptosis and necrosis) and active secretion (integrated in exosomes)^16–18^ and is highly fragmented^19^. CNV may additionally contribute to its high fragmentation. Moreover, the majority of cfDNA originates from leukocytes that mask the small fraction of tumor-derived cfDNA in peripheral blood^16,17^.

Previously, we established an efficient method to detect CNV in serum cell-free DNA (cfDNA) of cancer patients using MLPA^20–22^. MLPA is a semi-quantitative technique for determining the relative CNV, including copy number gain and loss of tumor suppressor genes and oncogenes in a multiplex PCR^22^. In addition, MLPA with its custom-developed data analysis software represents an easy, rapid and inexpensive method without the need of complex statistics. The present study aimed to evaluate CNV of various oncogenes and tumor suppressor genes in primary tumor tissue, cfDNA and lymph node metastasis; and to compare CNV profiles in serum with those in primary tumor and lymph node metastasis. Tumor progression is accompanied by additional genetic alterations^23^, and therefore CNV may be more present in genomic DNA of lymph node metastasis compared to primary tumor tissue. In this respect, we hypothesized that although primary tumor and lymph node metastasis show similar CNV profiles, we might detect more presence of CNV in lymph node metastasis. Since DNA is released into the blood circulation among others from primary tumor and metastasis by cell death, serum may reflect the CNV profiles from primary tumor and metastasis. This prompted us to hypothesize that CNV profiles of cfDNA from primary tumor and lymph node metastasis can be detected in serum.

## Results

### Copy number variations in primary tumor, serum and lymph node metastasis

**Figure 1:**
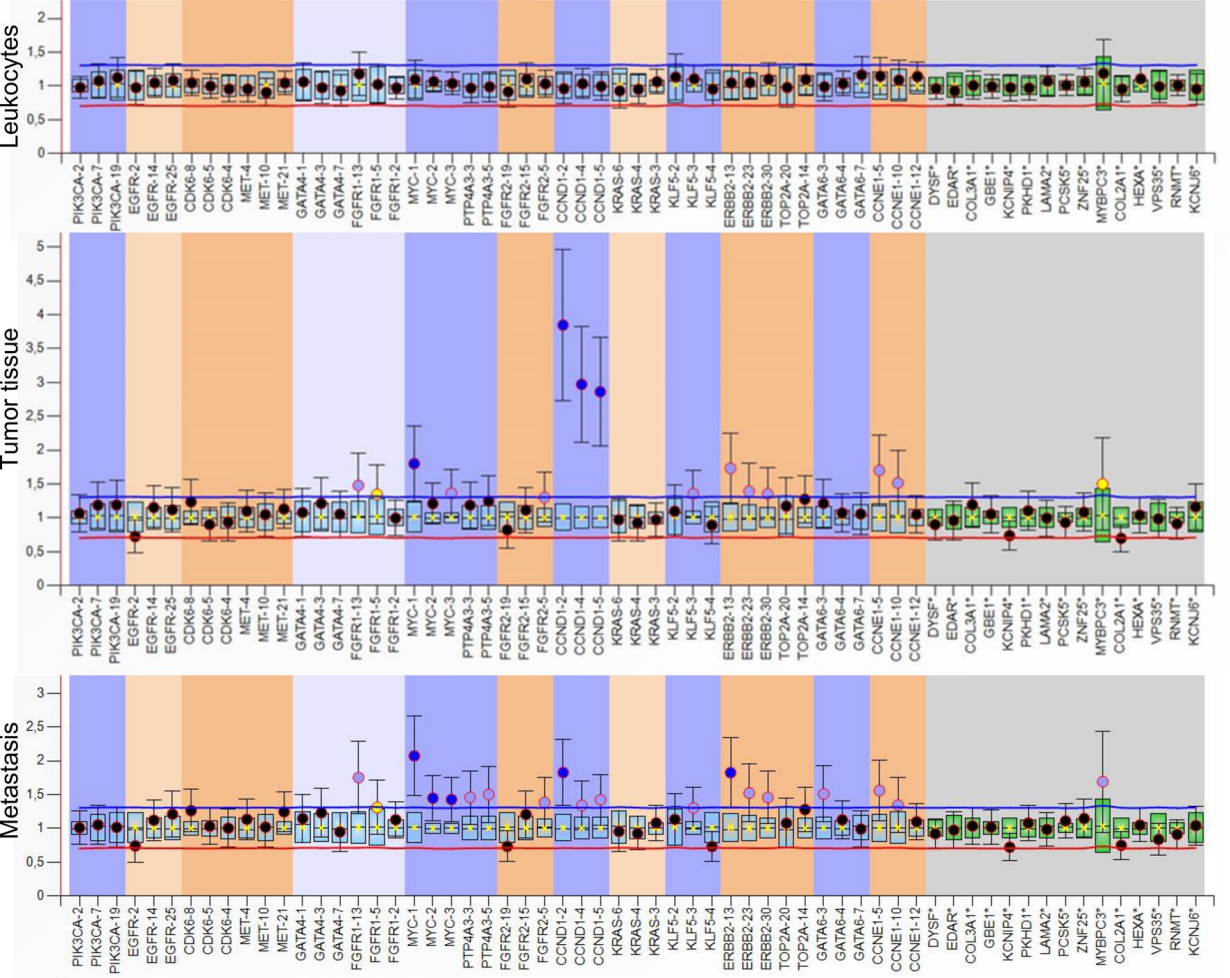
Example of CNV in primary tumor and lymph node metastasis of one patient. The box plot shows data of leukocytes (reference), primary tumor and lymph node metastasis (as calculated by Coffalyser.Net software). The DNA probes are arranged by chromosomal locations. The target-specific probes have a blue and orange background in different hues (left), whereas the reference probes have a grey background (right). Only the dark blue points indicate significant CNV gains, whereas light blue and yellow points are ambiguous and not considered. As expected the leukocyte DNA does not show any CNV. The data were calculated by intra- and inter-sample comparisons. Intra-sample normalization was performed by dividing the fluorescence signal of each target-specific probe by the signal of every single reference probe in this probe. The median of all these ratios of this probe is the normalization constant. Subsequently, inter-sample comparison was performed by dividing the normalization constant of each probe of this sample by the average normalization constant of all reference (leukocyte) samples.

In the present study, we analyzed 46 chromosomal regions containing 2–3 exons of 16 tumor-associated genes (Table S1) in tumor tissue, serum and lymph node metastasis of 72 UCB patients for CNV using the MLPA assay. In total, 29 (40.3%) patients were excluded due to insufficient amounts of the primary tumor, which impeded our analysis, resulting in 43 (59.7%) patients available for analyses. Table 1 summarizes the clinico-pathologic features of the study cohort. Patients' median age was 71 years, and 32 (74.4%) patients were male. As expected, leukocyte DNA did not show any CNV, and was used for data normalization. Figure 1 shows an exemplary box plot for CNV evaluation in primary tumor, lymph node metastasis and leukocytes (control) of a single patient who harbored CNV in both tissues.

**Table 1:** Descriptive characteristics of 43 urothelial carcinoma of the bladder patients treated with radical cystectomy and bilateral lymphadenectomy.

**Age (years)**	
Range, median	50–86, 71
**Gender [n (%)]**	
Male	32 (74.4)
Female	11 (25.6)
**Clinical tumor stage [n (%)]**	
cTa, cTis	2 (4.9)
cT1	6 (14.6)
cT2	32 (78.0)
cT3	1 (2.4)
**Clinical tumor grade [n (%)]**	
cG2	4 (9.8)
cG3	37 (90.2)
**Intravesical chemo- and/or immunotherapy prior to RC [n (%)]**	
No	33 (80.5)
Yes	8 (19.5)
**Number of TURB prior to RC**	
Range, median	1–5, 1
**Days between last TURB and RC**	
Range, median	7–480, 45
**Pathologic tumor stage [n (%)]**	
pT0, pTa, pTis	1 (2.3)
pT1	3 (7.0)
pT2	17 (39.5)
pT3	12 (27.9)
pT4	10 (23.3)
**Combined tumor stage [n (%)]**	
Localized (pT ≤ 2)	21 (48.8)
Advanced (pT3–4)	22 (51.2)
**Combined disease stage [n (%)]**	
≤ pT2 and pN0	19 (44.2)
≥ pT3 or pN1-3	24 (55.8)
**Pathologic tumor grade [n (%)]**	
G3	42 (97.7)
**Concomitant carcinoma in situ [n (%)]**	
Absent	23 (53.5)
Present	20 (46.5)
**Lymphovascular invasion [n (%)]**	
Absent	29 (67.4)
Present	14 (32.6)
**Microvessel invasion [n (%)]**	
Absent	37 (86.0)
Present	6 (14.0)
**Lymph node status [n (%)]**	
pN0	27 (62.8)
pN1–3	16 (37.2)
**Number of lymph nodes removed**	
Range, median	0–44, 12
**Soft tissue surgical margin status [n (%)]**	
Negative	35 (81.4)
Positive	8 (18.6)
**Urothelial carcinoma histology [n (%)]**	
Pure UCB	28 (65.1)
Presence of squamous cell differentiation	7 (16.3)
Presence of non-squamous cell differentiation	8 (18.6)
**Presence of incidental prostate cancer in the RC specimen [n (%)]**	
No	23 (53.5)
Yes	20 (46.5)
**Adjuvant chemotherapy [n (%)] **	
Not administered	29 (67.4)
Administered	14 (32.6)
**Adjuvant chemotherapy regimen [n (%)]**	
Cisplatin-based	6 (14.0)
Carboplatin-based	8 (18.6)

As shown in Table 2, 42 primary tumor tissues were available from 43 patients, while only metastasis tissue and serum were available from the one remaining patient. In total, 35 (83.3%) patients harbored CNV in the primary tumor. In the majority of UCB patients, CNV was detected in MYC and CCND1 in 27 (64.3%) patients and 17 (40.5%) patients, respectively. In the primary tumor, 15 (35.7%), 11 (26.2%), 4 (9.5%) and 4 (9.5%) patients harbored amplifications in all 3 exons of MYC, CCND1, ERBB2 and CCNE1, respectively. In each case, 2 patients (4.8%) harbored CNV in all 3 exons of EGFR and FGFR1, and only one patient (2.4%) had CNV in both exons of TOP2A, suggesting that the whole genes were possibly amplified in the primary tumor. Particularly, 4 (9.3%), 2 (4.7%) and one (2.3%) patient had even the copy number gain in all 3 exons of MYC together with the 3 exons of CCND1, ERBB2 or CCNE1, respectively. In each case, only one (2.3%) patient had CNV in all 3 exons of CCND1 and ERBB2 in the corresponding primary tumor and serum, while 2 (4.7%) patients had CNV in all 3 exons of CCND1 in the corresponding primary tumor and lymph node metastasis. The CNV in all 3 exons of MYC could not be found in the corresponding specimens. A heterogeneous CNV profile in serum cfDNA was revealed in 26 of 38 (68.4%) patients, with a low CNV (usually 0.5 to 2 fold), indicating that tumor cfDNA in serum was masked by normal wild type cfDNA. CNV in all 3 exons of MYC in the serum of one (2.6%) patient was not detected in primary tumor and lymph node metastasis. However, CNV in all 3 exons of CCND1 and ERBB2 in serum of one (2.6%) patient, respectively, were present in the primary tumor. Only 8 (42.1%) of 19 UCB patients harbored CNV in their lymph node metastasis. Lymph node metastasis of 3 (15.8%), 2 (10.5%) and 2 (10.5%) patients exhibited amplifications in all 3 exons of ERBB2, CCND1 and MYC, respectively. One (5.3%) patient had amplifications in all 3 exons of the 3 genes plus the both exons of TOP2A, which is localized together with ERBB2 on chromosome 17 (Table 2).

**Table 2:** CNV in primary tumor, serum and lymph node metastasis of 43 UCB patients treated with RC.

Gene		PIK3CA			EGFR			MET			GATA4			FGFR1			MYC			PTP4A		FGFR2			CCND1			KRAS			KLF5			ERBB2			TOP2A		GATA6			CCNE1			Total per patient	
Exon		2	7	19	2	14	25	4	10	21	1	3	7	13	5	2	1	2	3	3	5	19	15	5	2	4	5	6	4	3	2	3	4	13	23	30	20	14	3	4	7	5	10	12	Exon	Gene
Chromosome		3			7			7			8			8			8			8		10			11			12			13			17			17		18			19				
Patient	Type	Copy number variations																																												
1	T																**2**	**2**	**2**															2											4	1
	S																2																												1	
2	T																**2**	**2**	**2**						2							1.5													5	1
	S																												0.5																1	
3	T																**3**	**3**	**3**	**2**	**3**	0.5									2.5	3							2.5	2					10	2
	S																												0.5																1	
4	T																**2**	**1.5**	**1.5**						2							1.5		**2**	**1.5**	**1.5**						2	2		10	2
	M																																													
	S																												0.5																1	
5	T																**1.5**	**1.5**	**1.5**													1.5										**2**	**2**	**1.5**	7	2
	S																																						1.5						1	
6	T																		1.5													1.5													2	
	M																																													
	S																												0.5																1	
7	T																**2**	**1.5**	**1.5**						2							2		2											6	1
	M																																													
	S																												0.5																1	
8	T																**3**	**1.5**	**1.5**			2																				2	2		6	1
	M																																													
	S																																													
9	T																3								5	5	4															2.5	2.5		6	1
	M																																													
	S																																													
10	T													**2.5**	**2**	**2**																		2											4	1
	M																																													
	S																																													
11	T				**3**	**4**	**4**							2																		1.5													5	1
	M																																													
	S																												0.5																1	
12	T																2								**4**	**3**	**3**																		4	1
	M																																													
	S																**2**	**1.5**	**1.5**						2									2											5	1
13	T																																													
	M																																													
	S																												0.5																1	
14	T																3								**6**	**6**	**5**																		4	1
	S																			0.5																									1	
15	T																**2**	**1.5**	**1.5**						**3**	**2**	**2**																		6	2
16	T																	2	2																										2	
	M																																													
	S																																													
17	T																**2**	**2**	**2**					2	**2**	**2**	**2**							2					2						9	2
	M																																													
	S									1.5																													2						2	
18	T																								**3.5**	**4.5**	**4**																		3	1
	M																**3**	**3**	**3**		3				**5**	**5**	**5**					2.5	2	**4**	**3.5**	**3**	**3**	**3**							14	4
	S																																													
19	T										0.5		0.5																																2	
	M										0.5																																		1	
	S																																													
20	T																	2	2						**4**	**4**	**4**																		5	1
	M																		2						**4**	**4**	**4**																		4	1
21	M										0.5																																		1	
	S																												0.5																1	
22	T																**2.5**	**2**	**2**						**5.5**	**3**	**3**					2								2					8	2
	S									1.5																																			1	
23	T											2					**2**	**2**	**2**					2	**9**	**9**	**8**					2	2	2											11	2
	S																								**2**	**2**	**2**																		3	1
24	T										0.5					0.5	**5**	**5**	**5**															**3**	**2.5**	**2.5**									8	2
	S																																													
25	T																																													
	S																											0.5	0.5																2	
26	T																2								**5**	**3**	**3**																		4	1
	S									1.5	0.5																																		2	
27	T																		2															2	2										3	
	S																																													
28	T																**2**	**1.5**	**1.5**																										3	1
	S			2		2																			2																				3	
29	T																																													
	M										0.5																																		1	
	S																								2											2			2						3	
30	T																**5**	**1.5**	**1.5**						2																				4	1
	S																								2														2						2	
31	T																																													
32	T																																													
	S																																													
33	T													**2**	**2**	**2**									**3**	**3**	**3**							2	2										8	2
	S			2							0.5																																		2	
34	T																															2													1	
	S									2																													2						2	
35	T																																	**2.5**	**2**	**2**									3	1
36	T																		2															**3**	**2**	**2**	**2**	**2**							6	2
	S																				2													**3**	**2**	**2**		2							5	1
37	T																																													
38	T																																													
	M						0.5										**2**	**2**	**2**																						0.5				5	1
	S																																													
39	T																		2													2													2	
	S																																													
40	T										0.5			0.5	0.5																										0.5	**3.5**	**3.5**	**3**	7	1
	S									1.5																													2						2	
41	T																	2	2													2		2							0.5	**2.5**	**2.5**	**2**	8	1
	S																																													
42	T				**7**	**6**	**6**											2	2							2	2							2.5	2							**2**	**2**	**2**	12	2
	M																	2	2													3		**7**	**7**	**6.5**									6	1
	S									1.5																																			1	
43	T																**3.5**	**3.5**	**3.5**		2					2	7							3											7	1
	M																																	**7**	**6**	**5**	4.5								4	1
	S									1.5																																			1	
Total per exon	T				2	2	2				3	1	1	4	3	3	19	19	23	1	2	2		2	15	13	13				1	12	1	14	7	4	1	1	2	2	2	7	7	4		
	M						1				3						2	3	4		1				2	2	2					2	1	3	3	3	2	1			1					
	S			2		1				7	2						2	1	1	1	1				5	1	1	1	9					2	1	2		1	6							

Bold values refer to the copy number variation of all investigated (2 or 3) exons of the gene.

*PIK3CA* phosphatidylinositol 3-kinase catalytic subunit alpha, *EGFR* epidermal growth factor receptor, *MET* mesenchymal-epithelial transition, *GATA4* GATA binding protein 4, *FGFR1* fibroblast growth factor receptor 1, *MYC* myelocytomatosis, *PTP4A3* protein tyrosine phosphatase 4A3, *CCND1* cyclin D1, *KLF5* Kruppel-like Factor 5, *ERBB2* Erb-b2 receptor tyrosine kinase 2, *TOP2A* DNA topoisomerase II alpha, *GATA6* GATA binding protein 6, *CCNE1* cyclin E1, *T* tumor, *M* metastasis, *S* serum.

### Associations of copy number variations with clinico-pathologic UCB characteristics

Table 3 presents CNV in various genes and their association with clinico-pathologic UCB characteristics: In the primary tumor, CNV in FGFR1 and ERBB2 were associated with variant histology (p ≤ 0.027); CNV in KLF5, PTP4A, MYC and ERBB2 with pathologic tumor stage (p ≤ 0.038); CNV in ERBB2 exon 13 and exon 30 with advanced pathologic tumor stage (p ≤ 0.048); CNV in ERBB2 with LVI (p ≤ 0.048); CNV in GATA4, MYC, KLF5, ERBB2 with MVI (p ≤ 0.045); CNV in GATA4 and TOP2A with a positive soft tissue surgical margin (STSM, p ≤ 0.039); CNV in ERBB2 and CCNE1 with the presence of incidental prostate cancer (p ≤ 0.029).

**Table 3:** Associations of CNV with clinico-pathologic UCB characteristics in 43 UCB patients treated with RC.

Parameters	Clinical tumor stage	Clinical tumor grade		Urothelial carcinoma histology	Combined tumor stage	Combined disease stage		Pathologic tumor stage	Lymph node status	Number of lymph nodes removed	LVI	MVI		STSM		Presence of incidental prostate cancer		Recurrence	Survival
Source	Serum	Serum	Met	Tumor	Tumor	Tumor	Met	Tumor	Met	Tumor	Tumor	Tumor	Serum	Tumor	Serum	Tumor	Serum	Tumor	Tumor
Genes_Exon																			
MET_4		n.a.																	
MET_10		n.a.																	
MET_21		**0.003**																	
GATA4_1	**0.016**											0.334		0.518					
GATA4_3	n.a.											**0.014**		0.628					
GATA4_7	n.a.											0.683		**0.039**					
FGFR1_13				0.487															
FGFR1_5				0.223															
FGFR1_2				**0.014**															
MYC_1	**0.016**									**0.013**		0.803							
MYC_2	0.172									0.521		**0.045**							
MYC_3	0.172									0.977		0.133							
MYC_1,2,3								**0.038**											
PTP4A3_3	**0.001**		n.a.					**0.005**							**0.035**				
PTP4A3_5	n.a		**0.005**					0.075							**0.035**				
CCND1_2										**0.045**			0.465				**0.018**		
CCND1_4										**0.035**			**0.004**				0.317		
CCND1_5										**0.035**			**0.004**				0.317		
CCND1_2,4,5										**0.025**									
KRAS_6	**0.0001**														**0.035**				
KRAS_4	0.285														0.740				
KRAS_3	n.a.														n.a				
KLF5_2			n.a					**0.005**				0.683							
KLF5_3			0.071					0.445				0.783							
KLF5_4			**0.005**					0.475				**0.014**							
ERBB2_13			**0.001**	**0.027**	**0.018**	**0.030**		**0.027**			0.062	0.065			0.243	**0.005**		**0.008**	
ERBB2_23			**0.001**	**0.024**	0.275	0.075		0.547			**0.012**	**0.019**			**0.035**	**0.029**		0.086	
ERBB2_30			**0.001**	**0.004**	**0.048**	0.059		0.078			**0.048**	**0.0001**			0.243	0.255		**0.006**	
ERBB2_13,23,30																		**0.006**	
TOP2A_20			**0.0001**											**0.039**	n.a.			**0.002**	
TOP2A_14			**0.005**											**0.039**	**0.035**			**0.002**	
CCNE1_5																**0.029**			
CCNE1_10																**0.029**			
CCNE1_12																**0.029**			
CCNE1_5,10,12																**0.029**			
n Exons			**0.037**				**0.019**		**0.019**							**0.003**		0.303	0.333
n Genes			**0.009**				0.087		0.087							**0.015**		**0.043**	**0.032**

Only those genes with CNV are shown. The significant p-values are in bold. The insignificant p-values are also shown if one or two exons of the same gene display a significant p-value.

*EGFR* epidermal growth factor receptor, *MET* mesenchymal-epithelial transition, *GATA4* GATA binding protein 4, *FGFR1* fibroblast growth factor receptor 1, *MYC* myelocytomatosis, *PTP4A3* protein tyrosine phosphatase 4A3, *CCND1* cyclin D1, *KLF5* Kruppel-like Factor 5, *ERBB2* Erb-b2 receptor tyrosine kinase 2, *TOP2A* DNA topoisomerase II alpha, *CCNE1* cyclin E1, *TURB* transurethral resection of bladder, *RCE* radical cystectomy, *ConcCIS* concomitant carcinoma in situ, *N* lymph node, *MVI* microvessel invasion, *STSM* soft tissue surgical margin, *PCa* prostate cancer, *n* number.

In serum, CNV in CCND1 was associated with MVI (p = 0.004); CNV in PTP4A, KRAS, ERBB2, TOP2A with positive STSM (p ≤ 0.035); CNV in CCND1 with the presence of incidental prostate cancer (p = 0.018).

### Outcomes according to copy number variations

The median follow-up was 15.5 months (IQR: 4.5; 22.8). Actuarial two-year recurrence-free, cancer-specific and overall survival estimates were 68% ± 8% (standard error), 89% ± 6% and 82% ± 8% respectively.

**Figure 2:**
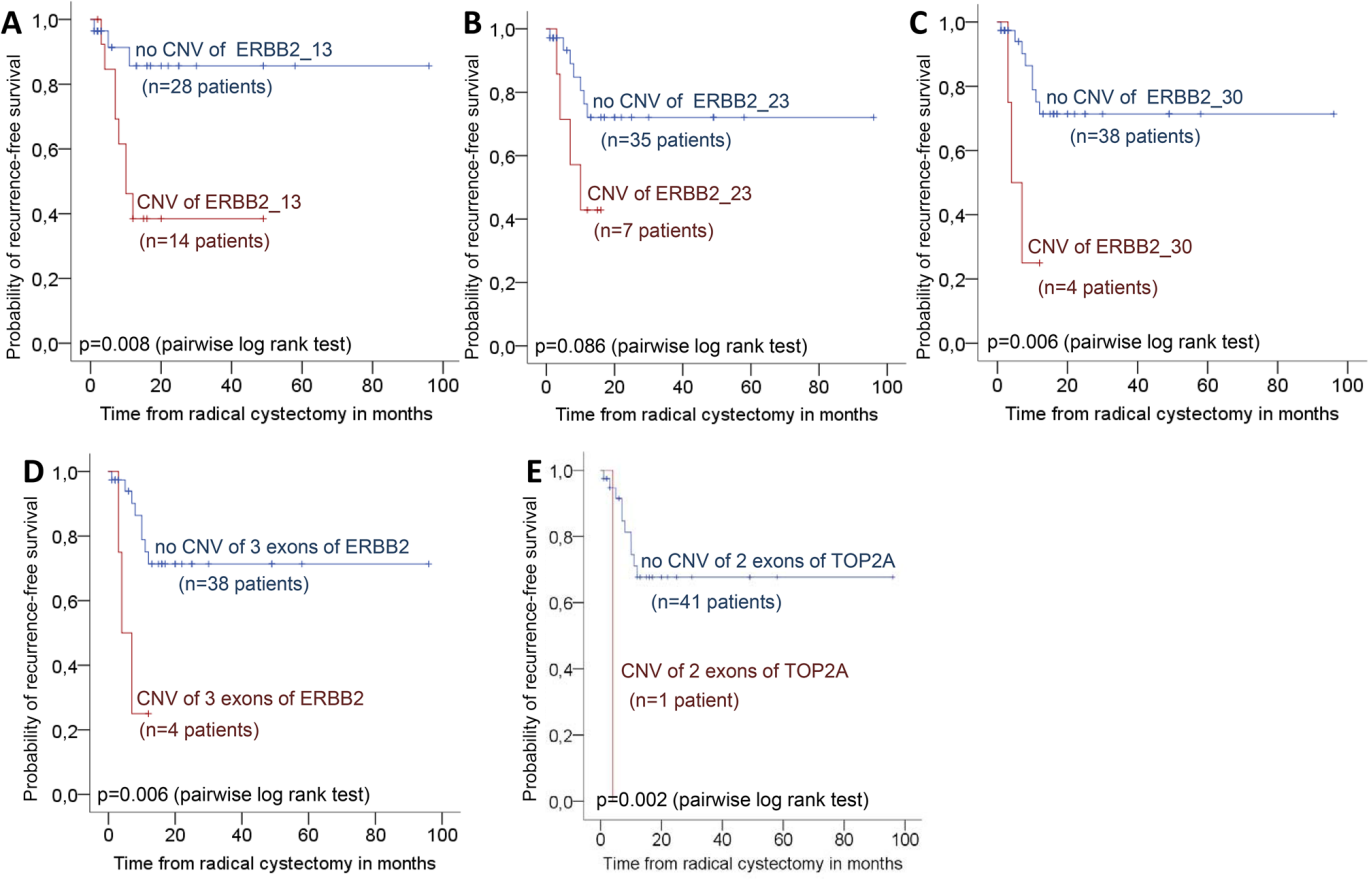
Kaplan–Meier plots of recurrence-free survival stratified by CNV in ERBB2 (**A**–**D**) and TOP2A (**E**) in 43 UCB patients treated with RC. Top curves (in blue) show UCB patients with no CNV (no genomic aberrations), and bottom curves (in red) show patients with CNV comprising DNA gains in the 13th exon (ERBB2_13. **A**), 23th exon (ERBB2_23, **B**), 30th exon (ERBB2_30, **C**) and all 3 exons patients of ERBB2 (**D**), as well as in both exons of TOP2A (**E**).

In Kaplan–Meier analyses there was no difference in cancer-specific and overall survival according to the CNV status in primary tumor, serum and lymph node metastasis. However, patients with CNV in exon 13, exon 30 and all 3 exons of ERBB2 in the primary tumor had significantly reduced recurrence-free survival, compared to patients without CNV in ERBB2 (pairwise p-values ≤ 0.008; Fig. 2A–D). In univariate Cox regression analysis, CNV in exon 13 (HR: 4.983, p = 0.018), exon 30 (HR: 5.374, p = 0.015) and all 3 exons (HR: 5.374; p = 0.015) of ERBB2 in the primary tumor were risk factors for disease recurrence.

In addition, one patient with CNV in exon 20 and 14 of TOP2A in the primary tumor had reduced recurrence-free survival, compared to the remaining patients without CNV in TOP2A (pairwise p = 0.002, Fig. 2E). In univariate Cox regression analysis, CNV in both exons of TOP2A in the primary tumor was a risk factor for disease recurrence (HR: 17.134, p = 0.021).

## Discussion

For the first time, the present study analyzed CNV in genomic DNA derived from primary tumor and lymph node metastasis, as well as in cfDNA from serum of UCB patients treated with RC, using MLPA. We found that MLPA is an efficient method for the detection of CNV in genomic DNA from primary tumor and metastasis, as well as cfDNA from serum. MLPA is a high throughout analysis, allowing simultaneous evaluation of up to 96 samples, with results being available within 24 h. Therefore, MLPA represents a promising tool for further investigations of the genomic landscape and metastatic cascade of UCB patients. We found most CNV in DNA of primary tumor, and to a lesser extent in lymph node metastasis and in cfDNA from serum. Thus, we have to reject our hypothesis. The lower extent of CNV in cfDNA may be due to dilution of tumor-derived cfDNA by wild type cfDNA in the blood of cancer patients that camouflages the detection of CNV in tumor-derived cfDNA. Thus, the heterogeneous CNV profile may be due to the low prevalence of tumor-derived cfDNA in the serum of UCB patients^18,24^. The lower extent of CNV in genomic DNA of lymph node metastasis may be due to limited amount of metastatic tumor tissue in lymph nodes, possibly influencing the efficiency of MLPA.

In the primary tumor, most CNV were found in MYC, CCND1, ERBB2 and CCNE1. Importantly, CNV in these genes were associated with aggressive clinico-pathologic UCB features. Only one patient had CNV in all 3 exons of CCND1 and ERBB2 in both, serum and primary tumor. In contrast, CNV in all 3 exons of MYC were exclusively detected in the serum of one patient, but not in the primary tumor. Although it remains speculative, it is possible that in this patient CNV in all 3 exons of MYC may not originate from the primary tumor, but rather from circulating tumor cells, which have a deleterious impact on outcome in UCB^25^. In UCB, alterations of chromosome 8, especially DNA deletions of the 8p arm and gains of the 8q arm, belong to the most frequent cytogenetic changes^26,27^. MYC is located on 8q24.21, and displayed copy number gains in all 3 exons in our study. Its amplification and overexpression have been described as main events, by which MYC is deregulated in various cancer entities^28,29^. Deregulation of MYC activity contributes to cancer progression, metastasis and resistance to therapy. MYC is a transcription factor and activates the expression of multiple genes that encode for proteins involved in cell proliferation, differentiation, adhesion and survival^28,30^. Using high-throughput tissue microarray, Zaharieva et al.^31^ showed that MYC gains are associated with genetically unstable UCB which is characterized by high histologic grade and/or invasive growth. Conconi et al.^9^ applied array-CGH and detected that bladder cancer stem cell (CSC) subpopulations retained CNV in MYC. These researchers postulated MYC to be a therapeutic target for bladder CSC subpopulations. We found that 15 UCB patients harbored CNV in all 3 exons of MYC gene in the tumor tissue. In addition, some patients harbored CNV in all 3 exons of MYC together with the 3 exons of CCND1, ERBB2 or CCNE1, respectively. Our analyses in primary tumor showed that CNV in all 3 exons and CNV in the second exon of MYC were associated with unfavorable UCB features, e.g. pathologic tumor stage and MVI. In addition, CNV in exons of other genes, including CCND1, FGFR1 ERBB2, KLF5, PTP4A, GATA4, TOP2A and CCNE1 were associated with aggressive clinico-pathologic UCB characteristics, e.g. presence of variant histology, LVI and positive STSM. Previously, it has been suggested that KLF5 facilitates angiogenesis and that KLF5 might represent a therapeutic target in UCB^32^. Correspondingly, GATA4 may potentially represent a therapeutic target, since whole exome sequencing showed that UCB patients with high expression of GATA4 have worse survival compared to patients with low GATA4 expression^33^. CCNE1 is located on 19q12 and has the same functions as CCND1. It induces S phase entry of the cell cycle and specifically interacts with CDK2^34^. The molecular signature of CCNE1 defined by CNV and expression changes has been reported to be an independent risk factor for disease progression in UCB patients. Moreover, gene network and upstream regulator analyses revealed that disease progression is potentially mediated by the CCND1-CCNE1-SP1 pathway^9,35,36^. Our analyses showed that in primary tumor CNV in CCNE1 is associated with presence of incidental prostate cancer.

Watters et al.^29^ showed that the majority of bladder carcinomas are polysomic for chromosome 8 and 11. These characteristics were reflected by the high CNV detected in MYC and CCND1. Amplifications of both genes have been reported to be associated with muscle-invasive UCB^29^. CCND1 regulates the cell cycle transition from G1 to S phase by the cyclin-dependent kinases CDK4 and CDK6. Its overexpression results in a dysregulated CDK activity followed by a rapid cell growth^37^. CCND1 is located on 11q13.2, and amplification of this chromosomal band is a common event in cancer^38^. In our investigations on DNA of the primary tumor, we found prominent copy number gains in all 3 exons of CCND1, in line with the findings by Chekaluk et al., who also used MLPA^39^, and Weltman et al., who used array-CGH^36^.

We found CNV in ERBB2 in DNA of primary tumor and lymph node metastasis. ERBB2 is a member of the ErbB receptor family, which is often overexpressed, amplified or mutated in various cancer types. ERBB2 regulates epithelial-mesenchymal transition (EMT), migration and tumor invasion by modulating extracellular matrix (ECM) components^40^. In metastases, a considerable heterogeneity in centromere 17 and CNV in ERBB2 located on 17q12 have been described, supporting genomic instability of these cells^41^. Our analyses on primary tumor showed that CNV in different exons of ERBB2 were associated with aggressive UCB features, e.g. presence of variant histology, pathologic tumor stage, LVI and MVI, and incidental prostate cancer. In addition, CNV in the exons of TOP2A, a topoisomerase which is also located on chromosome 17^42^, was associated with positive STSM. As TOP2A gene is located adjacent to the ERBB2 gene, it is frequently either co-amplified with or independent of ERBB2 in many cancer types, including UCB^43^. In addition, simultaneous amplification of both genes caused by different mechanisms was described in breast cancer, and contributed to a higher level of amplification of ERBB2^44^. In our cohort, patients with CNV in these genes in the primary tumor were at increased risk for disease recurrence. However, the patient number in the subgroup of patients with CNV in ERBB2 and TOP2A is very low. Thus, it has to be emphasized, that these results have to be interpreted very carefully. Although it remains speculative, patients with CNV in ERBB2 and CNV in TOP2A in the primary tumor and/or lymph node metastasis may benefit from HER2- or TOP2A-targeted therapy. Previously, a negative impact of ERBB2 amplifications on outcomes have been reported also in non-muscle invasive UCB^45,46^. Similar to our analyses, Kim et al.^47^ revealed that the levels of TOP2A expression are a risk factor for disease recurrence.

The present study is not devoid of limitations. A relevant number of patients was excluded from analyses, which might have influenced findings. The resulting total patient number was low and follow-up was limited. Thus, a multivariable analysis was not possible to identify independent risk factors for disease recurrence, cancer-specific and overall mortality. Nevertheless, our study remains the first for the analysis of CNV in genomic DNA from primary tumor, lymph node metastasis and tumor-derived cfDNA from serum using MLPA in UCB patients treated with RC. The MLPA assay, which was used in the present study, was not specifically manufactured or validated for analyses of UCB patients. However, we have previously shown that MLPA is an efficient method for the detection of CNV in UCB patients treated with RC^20^. The MLPA assay did contain sequences to analyze a limited number of genes, but of several exons; some genes that may have an important role in bladder cancer progression were not included in this assay, e.g. the androgen receptor, which may contribute to disease progression by EGFR signaling^48^. Lastly, since the amount of cfDNA was low, we could not perform single analyses on every chromosomal region by real-time Taqman PCR. Further studies are warranted to complete such evaluations in the future.

In conclusion, MLPA using genomic DNA derived from primary tumor tissue and lymph node metastasis, as well as cfDNA may offer a less time-consuming tool for the quick analysis of CNV in oncogenes and tumor suppressor genes in UCB. CNV are present in various genes of genomic DNA derived from primary tumor, lymph node metastasis and cfDNA from serum in UCB patients treated with RC. Most CNV were detected in all 3 exons of MYC, CCND1, ERBB1 and CCNE1. CNV in specific genes of genomic DNA derived from primary tumor, lymph node metastasis, and cfDNA are associated with unfavorable clinico-pathologic UCB features. In addition, patients with CNV in ERBB2 and TOP2A in the primary tumor are at increased risk for disease recurrence. Further studies in larger patient cohorts are necessary to validate our findings, and to evaluate whether these genes are suitable biomarkers for disease progression and candidates for targeted-therapy.

## Material and methods

### Patient cohort

The cohort of 72 UCB patients has been described in detail previously^20^: Briefly, patients were treated with RC and bilateral pelvic lymphadenectomy without neoadjuvant chemotherapy. Recurrent Ta, T1, or carcinoma in situ (CIS), refractory to transurethral resection of the bladder (TURBT) with or without intravesical immunotherapy or chemotherapy, or muscle invasive UCB were indications for RC. Preoperative staging consisted of computed tomography (CT) of the thorax and abdomen/pelvis, and bone scan and cranium imaging when clinically indicated. Exclusion criteria included metastatic disease at preoperative staging, a history of any other malignancy, previous systemic chemotherapy or radiation, and incomplete clinico-pathologic or follow-up data. Patients received adjuvant chemotherapy based on tumor stage, overall health status, renal function and patients' desire. Platinum-based adjuvant chemotherapy generally started within 90 days after RC. All experiments have been carried out in accordance with relevant regulations: all patients have signed written informed consent, and the study was approved by the local ethics committee "Universitätsklinikum Hamburg-Eppendorf; Ethik-Kommission der Ärztekammer Hamburg" (No. PV3962).

### Pathological evaluation

As described in detail previously^20^, the complete surgical RC specimen was inked, and multiple sections were obtained from the bladder and the tumor in addition to the regional lymph nodes and ureters. Tumor stage and nodal status were assessed according to the tumor, lymph node and metastasis (TNM) system. Tumor grade was assessed according to the 1998 World Health Organization (WHO) grading system^49^. Concomitant CIS was defined as the presence of CIS in conjunction with another tumor other than CIS alone. Lymphovascular invasion (LVI) was defined as the unequivocal presence of tumor cells within an endothelium-lined space without underlying muscular walls^50^. Micro-vascular invasion (MVI) was defined as the presence of tumor cells within a vessel with a vascular wall and red blood cells in the lumen^51^. A positive soft tissue surgical margin (STSM) was defined as the presence of tumor at inked areas of soft tissue on the RC specimen^52^. Presence of variant UCB histology was defined as the presence of UCB combined with any variant histology. Variant UCB histologies were classified corresponding to the WHO Classification of Tumors^53^. Incidental prostate cancer was defined as presence of prostate cancer in the RC specimens^54^.

### DNA extraction from tumor tissues

Unstained paraffin‐embedded primary tumor tissue and lymph node metastasis blocks were incubated at 50 °C for 30 min and de-paraffinized twice with xylol and 6 times with ethanol. After washing with water, the tumor areas were scrapped off from the slides with lysis buffer, as indicated by the slices stained with haematoxylin and eosin (Merck, Darmstadt, Germany). Genomic DNA was isolated using the Innu Prep DNA Micro Kit (Analytik Jena, Germany) and corresponding to the manufacturer´s recommendations. Briefly, the tumor tissues were incubated with 200 µl lysis buffer TLS and 20 µl proteinase K at 50 °C till the sample was completely lysed. After incubating the lysed samples at 90 °C for 60 min, they were supplemented with 200 µl Binding Solution TBS and added to spin filters. After washing the filters, DNA was eluted in 30 µl Elution Buffer (Analytik Jena).

### DNA extraction from serum and leukocytes

Preoperative blood samples were usually collected on the day prior to RC at a median of 39 days [interquartile range (IQR): 27; 61] after the preceding TURB. Serum was prepared from 6 ml whole blood by 2 centrifugation steps of 3000 g and 16,000 g each for 10 min. Leukocytes (reference) were extracted from 6 ml EDTA blood supplemented up to 50 ml with lysis buffer containing 0.3 M sucrose, 10 mM Tris–HCl pH 7.5, 5 mM MgCl2 and 1% Triton X100 (Sigma, Taufkirchen, Germany). Following incubation for 15 min on ice, the isolation and purification of the leukocytes were carried out by 2 centrifugation steps at 2500 *g*, at 4 °C for 20 min. cfDNA was extracted from 2 ml serum using the PME free-circulating DNA Extraction kit (Analytik Jena), while DNA was extracted from leukocytes using the Qiamp DNA Blood Mini kit (Qiagen, Hilden, Germany). These DNA extractions were carried out according to the manufacturer´s instructions and similar to the procedure as described above. Quantification and quality of the extracted cfDNA were determined spectrophotometrically using the NanoDrop Spectrometer ND-1000 (Thermo Fisher Scientific, Wilmington, DE, USA).

### MLPA assay

MLPA experiments were already described in our both previous studies^20,22^ and are briefly described in the following paragraphs, again: CNV were determined using 5 µl (50 ng) cfDNA and 5 µl of each (100 ng) leukocytes (reference), tumor tissues and metastasis DNA from 46 bladder cancer patients and the SALSA MLPA probemix SALSA MLPA Probemix P458-B1 kit (MRC Holland, Amsterdam, The Netherlands). This kit contains a probe mix of 46 sequences of 16 genes to be analyzed (Table S1), 15 reference genes (Table S2) and 9 quality control fragments (Table S3). According to the manufacturer’s instructions, 5 µl DNA samples were denatured at 98 °C for 5 min, and hybridized with 1.5 probemix and 1.5 µl MLPA buffer at 60 °C for about 18 h. Next day, the hybridization reaction was ligated with Ligase-65 master mix containing 25 µl water, 3 µl ligase buffer A, 3 µl ligase buffer B and 1 µl Ligase-65 enzyme at 54 °C for 15 min and at 98 °C for 5 min. Then, the MLPA reaction was mixed with polymerase master mix containing 7.5 µl water, 2 µl SALSA PCR primer mix and 0.5 µl SALSA polymerase. PCR was carried out at 95 °C for 30 s, at 60° for 30 s and at 72 °C for 60 s in 35 cycles, with a last step of 72 °C for 20 min on MJ Research PTC-200 Peltier Thermal Cycler (Global Medical Instrumentation, Ramsey, Minnesota, USA). During the PCR, all MLPA samples were amplified simultaneously using the same PCR primer pair, of which one PCR primer was fluorescently labelled.

### Capillary electrophoresis

For fragment analysis, 1.4 µl PCR was mixed with 0.6 µl 500-ROX size marker which served as an internal standard (ThermoFisher, Darmstadt, Germany) and 18 µl HiDi formamide (ThermoFisher). After heating at 86 °C for 3 min and cooling down at 4 °C, fragment separation was done by capillary electrophoresis on an automated ABI 3130 DNA analyzer (Applied Biosystems, Freiburg, Germany). Fragment length and fluorescence intensity were evaluated by the Coffalyser.Net software (MRC). The Coffalyser.Net software could be downloaded for free after buying the MLPA kit which contained the corresponding chromosomal regions referring to the software.

### Data normalization

As previously described^20^, data normalization was carried out by Coffalyser.Net analysis software (www.mlpa.com). It consists of 2 steps: intra- and intersample normalization. For intrasample normalization, within each sample, each probe peak was compared with the peaks of the reference probes. Reference probes located on various chromosomes detect sequences that are expected to have a normal copy number in all samples. The determined relative probe signals were then used for intersample normalization (Table S1). Final probe ratios were determined by comparing the relative probe peak in the cfDNA sample of interest with those of all leukocyte DNA samples. Leukocyte DNA samples are expected to have a normal copy number for both the reference and target probe. To avoid false positive data due to the quality and quantity of the serum cfDNA, only unambiguous values were used (Fig. 1), and PCR was repeated.

MLPA was carried out using the panel MRC kit following the provided protocol. Data analysis was carried out using the Coffalyser program. Raw data of all leukocytes with adequate quality was taken as reference for calculating the 95% confidential interval reference range for each probe (Fig. 1). For each patient, ratio charts of leukocytes, tumor, metastasis and serum were compared. Probes with increase or increase in more than two standard deviations to the mean reference value were identified as duplication or deletion.

### Follow-up regimen

Follow-up has been outlined in detail previously^20^: For the first year, patients were seen every 3 months, from the second to fifth years every 6 months, and annually thereafter. Diagnostic imaging of the abdomen including the urinary tract (e.g. ultrasonography and/or intravenous urography, CT of the abdomen/pelvis with intravenous contrast) and chest radiography were performed at least annually or when clinically indicated.

Disease recurrence was defined as local failure in the operative site, regional lymph nodes, or distant metastasis. Upper tract urothelial carcinoma was not considered as disease recurrence but metachronous tumor. Cancer-specific mortality was defined as death from UCB. Overall mortality was defined as death from any cause. The cause of death was determined by the treating physician, by chart review corroborated by death certificates, or by death certificates alone^55^. Perioperative mortality (i.e., death within 30 days of surgery) was censored at time of death for bladder cancer-specific survival analyses.

### Statistical analyses

The co-primary endpoints of the present study were disease recurrence, cancer-specific and overall mortality according to CNV profile in 16 tumor suppressor genes and oncogenes. The indicator variable (i.e., CNV) was analyzed as categorical variables. Associations between categorical variables were assessed using the Fisher exact and χ^2^-test. Differences in continuous variables were analyzed using the Mann–Whitney-U test (two categories) and the Kruskal–Wallis test (three or more categories). Recurrence-free, cancer-specific and overall survival probabilities were estimated using the Kaplan–Meier method and differences between groups were assessed using the Log rank statistic. Univariable Cox regression models assessed time to disease recurrence, cancer-specific and overall mortality. All tests are two-sided and a p-value of < 0.05 was set to be statistically significant. All analyses were performed with SPSS 20 (SPSS Inc., IBM Corp., Armonk, NY).

## Supplementary information

Supplementary Information
