# Supplementary material for: Copy number variations in primary tumor, serum and lymph node metastasis of bladder cancer patients treated with radical cystectomy
Source: Sci Rep. 2020 Dec 9;10:21562. doi: 10.1038/s41598-020-75869-x (PMC7725833; doi:10.1038/s41598-020-75869-x)
Supplement: Supplementary file 1 — Supplementary Information [file 41598_2020_75869_MOESM1_ESM.doc]

**Copy number variations in primary tumor, serum and lymph node metastasis of bladder cancer patients treated with radical cystectomy**

Armin Soave^1^, Lan Kluwe^2^, Hang Yu^1^, Michael Rink^1^, Philipp Gild^1^, Malte W. Vetterlein^1^, Philipp Marks^1^, Guido Sauter^3^, Margit Fisch^1^, Christian P. Meyer^1^, Tim Ludwig^1^, Roland Dahlem^1^, Sarah Minner^3^, Klaus Pantel^4^, Bettina Steinbach^4^, Heidi Schwarzenbach^4^*

^1^Department of Urology, University Medical Center Hamburg-Eppendorf, Hamburg, Germany

^2^Department of Neurology, University Medical Center Hamburg-Eppendorf, Hamburg, Germany

^3^Institute of Pathology, University Medical Center Hamburg Eppendorf, Hamburg, Germany

^4^Department of Tumor Biology, University Medical Center Hamburg-Eppendorf, Hamburg, Germany

***Corresponding author:**

Heidi Schwarzenbach, PhD

Department of Tumor Biology

University Medical Center Hamburg-Eppendorf

Martinistraße 52

20246 Hamburg, Germany

Phone: +49 40 7410 57494

Fax: +49 40 7410 56546

Email: [hschwarzenbach@uke.de](mailto:hschwarzenbach@uke.de)

**Table S1:** SALSA MLPA Probemix P458-B1 arranged according to chromosomal location

| **Genes** | **Chromosome** | **Exon** | **Chromosomal band** | **Length**  **(bp)** |
| --- | --- | --- | --- | --- |
| PIK3CA | 3 | 2 | 3q26.32 | 436 |
|  |  | 7 |  | 250 |
|  |  | 19 |  | 215 |
| EGFR | 7 | 2 | 7p11.2 | 449 |
|  |  | 14 |  | 283 |
|  |  | 25 |  | 332 |
| CDK6 | 7 | 8 | 7q21.2 | 364 |
|  |  | 5 |  | 426 |
|  |  | 4 |  | 478 |
| MET | 7 | 4 | 7q31.2 | 202 |
|  |  | 10 |  | 161 |
|  |  | 21 |  | 301 |
| GATA4 | 8 | 1 | 8p23.1 | 319 |
|  |  | 3 |  | 146 |
|  |  | 7 |  | 130 |
| FGFR1 | 8 | 13 | 8p12 | 358 |
|  |  | 5 |  | 178 |
|  |  | 2 |  | 484 |
| MYC | 8 | 1 | 8q24.21 | 166 |
|  |  | 2 |  | 463 |
|  |  | 3 |  | 406 |
| PTP4A3 | 8 | 3 | 8q24.3 | 276 |
|  |  | 5 |  | 232 |
| FGFR2 | 10 | 19 | 10q26.13 | 150 |
|  |  | 15 |  | 245 |
|  |  | 5 |  | 344 |
| CCND1 | 11 | 2 | 11q13.2 | 265 |
|  |  | 4 |  | 209 |
|  |  | 5 |  | 308 |
| KRAS | 12 | 6 | 12p12.1 | 124 |
|  |  | 4 |  | 197 |
|  |  | 3 |  | 399 |
| KLF5 | 13 | 2 | 13q22.1 | 456 |
|  |  | 3 |  | 385 |
|  |  | 4 |  | 227 |
| ERBB2 | 17 | 13 | 17q12 | 185 |
|  |  | 23 |  | 238 |
|  |  | 30 |  | 351 |
| TOP2A | 17 | 20 | 17q21.2 | 141 |
|  |  | 14 |  | 325 |
| GATA6 | 18 | 3 | 18q11.2 | 418 |
|  |  | 4 |  | 378 |
|  |  | 7 |  | 288 |
| CCNE1 | 19 | 5 | 19q12 | 271 |
|  |  | 10 |  | 172 |
|  |  | 12 |  | 391 |

**Table S2:** Reference probes arranged according to chromosomal location

| **Genes** | **Chromosome** | **Chromosomal band** | **Length**  **(bp)** |
| --- | --- | --- | --- |
| DYSF | 2 | 2p13 | 191 |
| EDAR | 2 | 2q13 | 156 |
| COL3A1 | 2 | 2q32 | 256 |
| GBE1 | 3 | 3p12 | 295 |
| KCNIP4 | 4 | 4p15 | 490 |
| PKHD1 | 6 | 6p12 | 113 |
| LAMA2 | 6 | 6q22 | 220 |
| PCSK5 | 9 | 9q21 | 337 |
| ZNF25 | 10 | 10p11 | 412 |
| MYBPC3 | 11 | 11p11 | 118 |
| COL2A1 | 12 | 12q13 | 499 |
| HEXA | 15 | 15q23 | 442 |
| VPS35 | 16 | 16q11 | 371 |
| RNMT | 18 | 18p11 | 469 |
| KCNJ6 | 21 | 21q22 | 137 |

**Table S3:** Nine quality control fragments generating amplification products between 64 and 121

**Length (nt) Name**

64-70-76-82 Q-fragments (Only visible with <100 ng sample DNA)

88-96 D-fragments (Low signal of 88 nt or 96 nt fragment indicates incomplete denaturation)

92 Benchmark fragment

100 X-fragment (X chromosome specific)

105 Y-fragment (Y chromosome specific)
